# The Role of 5-HT and DA Receptor Genes in Starvation-Induced Anxiety Behavior of *Portunus trituberculatus*

**DOI:** 10.3390/genes17060678

**Published:** 2026-06-10

**Authors:** Yangyang Lv, Wei Zhai, Yuanyuan Fu, Sixiang Wang, Lei Liu

**Affiliations:** 1 School of Marine Sciences, Ningbo University, Ningbo 315832, China; 2 Ningbo Institute of Oceanography, Ningbo 315832, China

**Keywords:** aggressive behavior, 5-hydroxytryptamine, maze, ethology, stress response

## Abstract

Background/Objectives: Intraspecific aggression and cannibalism severely reduce survival in aquaculture of the Japanese swimming crab, *Portunus trituberculatus*. Starvation can induce stress-related behavioral changes and alter aggressive behavior in animals, but its behavioral and neurochemical effects in crustaceans remain poorly understood. This study aimed to characterize starvation-associated behavioral changes in *P. trituberculatus* and to examine their associations with hemolymph serotonin (5-HT) levels, 5-HT1, 5-HT2, and DA1 expression, and aggressive behavior. Methods: Male *P. trituberculatus* (16 ± 1 g) were randomly assigned to starvation durations of 0–12 days. Starvation-associated behavioral responses were assessed with an underwater light–dark maze and open-field test, and video-recorded behavioral trajectories were analyzed. Hemolymph 5-HT was measured by ELISA. Aggressive behavior was recorded after pairing crabs showing different starvation-associated behavioral states. Expression of 5-HT1, 5-HT2, and *DA1* genes in multiple tissues was detected by RT-qPCR. Results: Starvation for 3–6 days significantly reduced time spent in the light arm and center zone, together with decreased hemolymph 5-HT levels. At 7–8 days, time spent in these zones increased and 5-HT levels increased, whereas these behavioral indices decreased again after 9 days. Starvation-associated behavioral changes were associated with reduced attack frequency and attack duration. Moreover, crabs showing starvation-associated behavioral changes showed altered expression of 5-HT1, 5-HT2, and *DA1* genes in muscle and eyestalk after aggressive encounters.

## 1. Introduction

Anxiety is characterized by stress-related behavioral and physiological responses and is often associated with depression [1]. In humans, anxiety can lead to serious consequences such as insomnia [2], cognitive impairment [3], and even suicide [4]. Most studies of anxiety-like responses have focused on vertebrates, but similar responses have also been reported in crustaceans with relatively simple nervous systems and these responses are regulated by serotonin (5-HT) [5]. Further studies have shown that anxiety can alter aggressive [6], exploratory [7], defense [8], and social and cognitive responses [9]. Anxiety-like responses are influenced by neurochemical factors, including serotonergic signaling and related pathways [10], as well as by environmental stressors [11]. In crustaceans, these behavioral responses can be induced by external stressors, and internal processes such as molting may also affect their expression [12]. Further studies suggest that anxiety may enhance vigilance toward potential threats and promote adaptive strategies that reduce mortality risk [13]. However, anxiety-like responses have rarely been studied in crabs, and there is still no clear criterion for identifying such responses in crab species. Therefore, establishing a behavioral assessment model for crabs is important for subsequent studies on behavioral regulation in crabs.

The Japanese swimming crab, *Portunus trituberculatus*, is a mariculture crab species of considerable economic importance in East Asia, but intraspecific competition greatly limits its sustainable cultivation. It has been reported that approximately 30% of juvenile *P. trituberculatus* suffer injuries or limb loss during antagonistic interactions, resulting in reduced competitive ability and survival [14]. Serotonin and dopamine are key neurotransmitters involved in aggression and play broad roles in the regulation of animal physiology and behavior [15]. Serotonergic and dopaminergic signaling is mediated by multiple receptor subtypes. In invertebrates, receptor genes homologous to vertebrate 5-HT1, 5-HT2, and 5-HT7 families have been reported, although their classification and functions may vary among species [16]. In decapod crustaceans, receptor subtypes such as 5-HT2B, 5-HT7, and DA2 have also been implicated in agonistic behavior [17]. Therefore, the present study focused on 5-HT1, 5-HT2, and DA1 as candidate genes, rather than representing a complete survey of all serotonergic and dopaminergic receptor families in *P. trituberculatus*. However, relatively few studies have investigated how serotonergic and dopaminergic signaling is associated with aggressive behavior in *P. trituberculatus*. Therefore, characterizing starvation-associated behavioral changes in this species and examining their associations with 5-HT/DA-related gene expression and aggressive behavior deserves particular attention.

In natural environments, starvation is a common stressor that triggers a range of physiological and behavioral responses [18]. In crustaceans and other aquatic animals, starvation can markedly affect survival, fitness, aggression, and cannibalism; in *P. trituberculatus*, short-term starvation can alter aggression and foraging activities [19]. Hunger has also been identified as an important factor promoting cannibalism [20], while starvation and body-size differences can enhance aggression and cannibalism [21]. In addition, hunger is closely associated with stress-related behavioral changes, and starvation-induced physiological and neurochemical changes may further influence aggressive behavior [22]. Therefore, investigating the effect of starvation on behavioral changes and aggression in crabs may provide a new perspective for understanding aggressive behavior and help improve the culture of this species.

In this study, to investigate starvation-associated behavioral changes and their relationship with aggression in *P. trituberculatus*, we first conducted experiments using an underwater light–dark maze [23] and an open-field test [24]. We quantitatively analysed the video-recorded data to determine temporal changes in behavioral indices [25]. We then examined changes in hemolymph 5-HT levels during starvation-associated behavioral responses. In addition, we evaluated the association between starvation-associated behavioral states and aggressive behavior by establishing attack-behavior groups according to the starvation time points at which reduced time spent in the light arm and center zone was observed. We also examined the expression of 5-HT1, 5-HT2, and DA1 in different tissues and combined molecular and behavioral data to examine associations between behaviors and key gene expression. This study will not only improve our understanding of behavioral adaptation in *P. trituberculatus*, but also provide a theoretical basis for behavioral trait evaluation and the breeding of low-aggression lines.

## 2. Materials and Methods

### 2.1. Material Crabs and Rearing Conditions

The *P. trituberculatus* were from a farm in Ningbo, Zhejiang Province, China. A total of 720 healthy male crabs (16 ± 1 g, with intact appendages and no visible external injury) from the same lineage were selected from the acclimated population. The experimental crabs were temporarily housed in aquariums connected to a recirculating water system and fed fresh clam meat daily during the rearing period. All crabs were raised in fresh seawater tanks maintained at 25 ± 0.5 °C and 20‰ salinity, with continuous aeration under a 12/12 h light/dark cycle. All experimental crabs were acclimated for at least one week before the experiment.

### 2.2. Behavioral Assays Under Starvation Stress

#### 2.2.1. Light and Dark Cross-Maze Experiment

**Figure 1:**
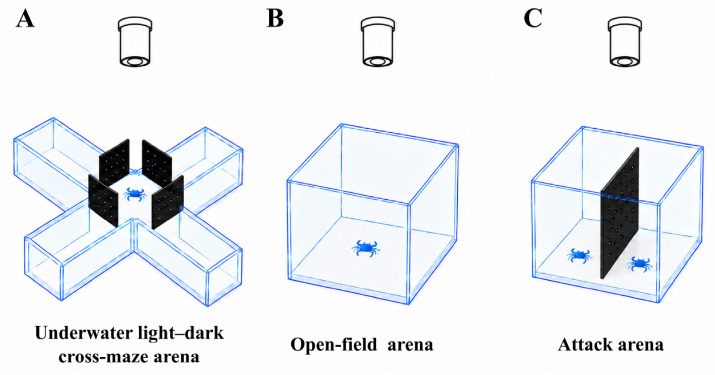
Schematic diagrams of the behavioral test arenas. (**A**) Underwater light–dark cross-maze arena; (**B**) open-field arena; (**C**) attack arena.

Starvation was used as the stressor, and the cross light–dark maze was used to evaluate starvation-associated behavioral changes and to determine the time points at which time spent in the light arm decreased. The underwater light–dark cross-maze arena was 25 cm × 25 cm × 10 cm (length × width × height; Figure 1A). The starvation duration was set from 0 to 12 days, resulting in 13 time-point groups. Independent groups of crabs were used for each starvation time point, and the same individuals were not repeatedly tested across different starvation durations. The 1-day starvation group was used as the behavioral reference group because crabs in this group showed stable locomotor activity and measurable time spent in the light arm and center zone. The 0-day fed control group was analyzed separately because recent feeding may affect locomotor activity and confound comparisons with starved groups. Each time-point group contained 12 crabs, and each crab was tested once as one biological replicate (*n* = 12 biological replicates per group). The interval between two adjacent time points was 24 h.

Each crab was first placed in a rectangular starting chamber in the center of the maze. The chamber was enclosed by removable opaque partitions with small holes to balance the internal and external water pressure. The partitions were removed one minute later, and the behavior of the crab was recorded for 10 min. After each experiment, the maze was flushed for 30 s to eliminate potential effects on subsequent individuals.

#### 2.2.2. Open-Field Experiment

The open-field arena was 30 cm × 30 cm × 20 cm (length × width × height; Figure 1B). The bottom area was divided into a center zone and a peripheral zone during video analysis. The starvation duration and grouping were the same as those used in the light–dark cross-maze experiment. During each trial, one crab was placed in the center of the bottom of the box. After 20 min of acclimation, its behavior was video-recorded for 30 min. The inside of the box was cleaned after each trial, as in the cross-maze test.

#### 2.2.3. Attack Experiment

According to the starvation time points at which decreased time spent in the light arm and center zone was observed, the starvation duration for the starvation-treated group was determined. The fighting arena was the same size as the open-field arena (Figure 1C). The experiment was divided into three groups with six replicates each: (1) CC, control–control group, with two 0-day fed control crabs paired; (2) SS, starved–starved group, with two starvation-treated crabs paired; and (3) CS, control–starved group, with one 0-day fed control crab and one starvation-treated crab paired. In each experiment, two male crabs of similar size were placed on opposite sides of the aquarium and separated by an opaque partition. The individuals were acclimated in the arena for 20 min. The partition was then removed, clam homogenate was injected, and fighting behavior was video-recorded during a 30 min observation interval. The videos were analyzed by blinded review and systematic scoring to record the attack behavior of each crab.

### 2.3. Hemolymph and Tissue Sampling

Hemolymph samples were collected after the behavioral tests for 5-HT measurement. For each starvation time point, hemolymph was collected from 12 crabs, with each crab used as one biological replicate (n = 12 biological replicates per group). For gene-expression analysis, muscle, eyestalk, ganglion, and gill tissues were collected from five individuals in each experimental group immediately after the aggressive-behavior assays, with each individual used as one biological replicate. Each sample was immediately frozen in liquid nitrogen for at least 5 min and stored at −80 °C until analysis.

### 2.4. Biochemical and Molecular Measurements

#### 2.4.1. Determination of Hemolymph 5-HT Concentration by ELISA

Crabs were anesthetized on ice. Hemolymph was withdrawn from the base of the swimming leg and mixed with an equal volume of pre-cooled 1× EDTA-K2 anticoagulant solution (catalog No. G0280; Solarbio, Beijing, China). The mixture was kept at 4 °C and centrifuged at 8000 r/min for 15 min at 4 °C. The supernatant was collected and stored at −80 °C until analysis. Hemolymph 5-HT concentration was measured using a 5-HT ELISA kit (Shanghai Kexing Biotechnology Co., Ltd., Shanghai, China).

#### 2.4.2. RT-qPCR Analysis

RT-qPCR was used to examine the expression of 5-HT1, 5-HT2, and DA1. The 5-HT1, 5-HT2, and DA1 sequences of *P. trituberculatus* or closely related species were retrieved from NCBI, including the *P. trituberculatus* 5-HT1 reference sequence (GenBank/RefSeq accession No. XP_045106432.1) [26]. Sequence alignment was performed using DNAMAN software (version 9.0; Lynnon Biosoft, San Ramon, CA, USA). Specific primers were designed from conserved regions using Primer Premier 6 software (PREMIER Biosoft International, Palo Alto, CA, USA) for 5-HT1, 5-HT2, DA1, and ribosomal protein L (RPL), which was used as an internal reference (Table 1). Primer specificity was evaluated using qPCR melting-curve data and in silico PCR against available *P. trituberculatus* transcript sequences. The predicted amplicon sizes were 207 bp for 5-HT1, 153 bp for 5-HT2, 171 bp for DA1, and 172 bp for RPL. Single melting peaks were observed for all primer pairs, supporting specific amplification (Supplementary Table S2).

Total RNA was isolated from the frozen tissue samples of different groups using TRIzol^®^ Plus RNA Purification Kit (Invitrogen, Carlsbad, CA, USA). First-strand cDNA was synthesized using PrimeScript™ RT reagent Kit with gDNA Eraser (Takara, Shiga, Japan). The synthesized cDNA was stored at −20 °C until RT-qPCR analysis.

RT-qPCR was performed using a LightCycler^®^ 96 real-time PCR system (Roche, Basel, Switzerland) with TB Green Premix Ex Taq™ (Tli RNaseH Plus) (Takara, Shiga, Japan). Each 20 μL reaction contained 10.0 μL TB Green Premix Ex Taq, 0.4 μL forward primer, 0.4 μL reverse primer, 2.0 μL cDNA template, and 7.2 μL DEPC-treated water. Each biological replicate was analyzed with three technical replicates. The amplification program was 40 cycles of 95 °C for 5 s, 60 °C for 30 s, and 72 °C for 30 s.

### 2.5. Data Analysis

The video-recorded data were analyzed and quantified using EthoVision XT 12.0 software (Noldus Information Technology, Wageningen, The Netherlands). Relative gene expression levels were calculated using the 2^−ΔΔCt^ method [27], with RPL as the internal reference gene. The stability of RPL expression was evaluated using Ct values across experimental groups (Supplementary Table S1). Experimental data were analyzed using SPSS 26.0 software. One-way ANOVA was used to compare differences among groups. Because independent groups of animals were used for each starvation time point, repeated-measures ANOVA was not applied. Tukey’s post hoc test was used for behavioral and hemolymph 5-HT datasets, whereas Duncan’s multiple range test was used for RT-qPCR gene-expression data. Data are presented as mean ± SEM. Stars in figures represent *p* < 0.05 (*), *p* < 0.01 (**), *p* < 0.001 (***), and *p* < 0.0001 (****), ns: not significant (*p* ≥ 0.05).

## 3. Results

### 3.1. Light and Dark Cross-Maze Experiment

**Figure 2:**
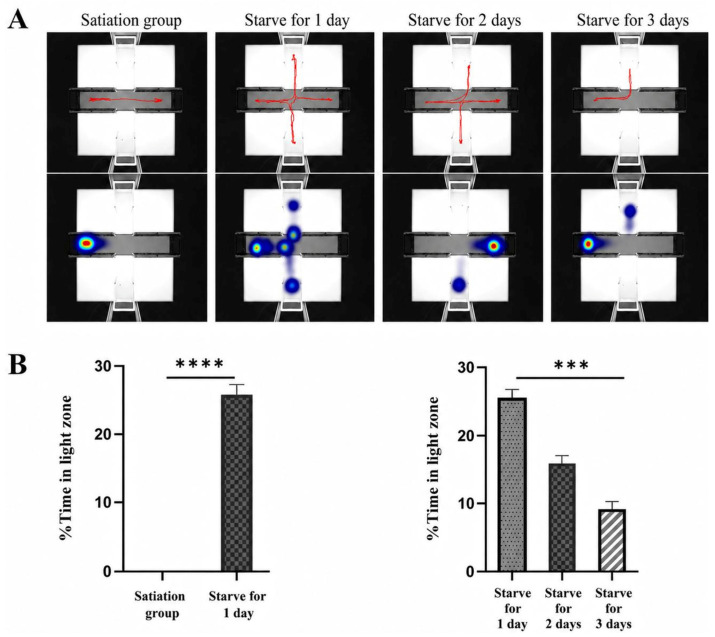
Starvation-associated behavioral responses of *P. trituberculatus* from 0 to 3 days. (**A**) Heatmap of movement trajectory; (**B**) percentage of time spent in the light arm. Asterisks in the figure represent *p* < 0.001 (***), and *p* < 0.0001 (****).

The underwater cross-maze experiment showed that starvation altered the time spent by *P. trituberculatus* in the light arm, and this behavioral response changed with prolonged starvation. In the 0-day fed control group, crabs mainly remained in the dark arm, which served as a dark refuge (Figure 2A). This pattern was significantly different from that in the 1-day starvation group, which was used as the behavioral reference group (*F*(1, 22) = 509.71, *p* = 1.04 × 10^−16^; Figure 2B). With increasing starvation time, the proportion of time spent in the light arm gradually decreased, showing a significant difference from the behavioral reference group after 3 days of starvation (*F*(1, 22) = 114.60, *p* = 3.45 × 10^−10^; Figure 2B), and reached its minimum value after 4 days of starvation.

**Figure 3:**
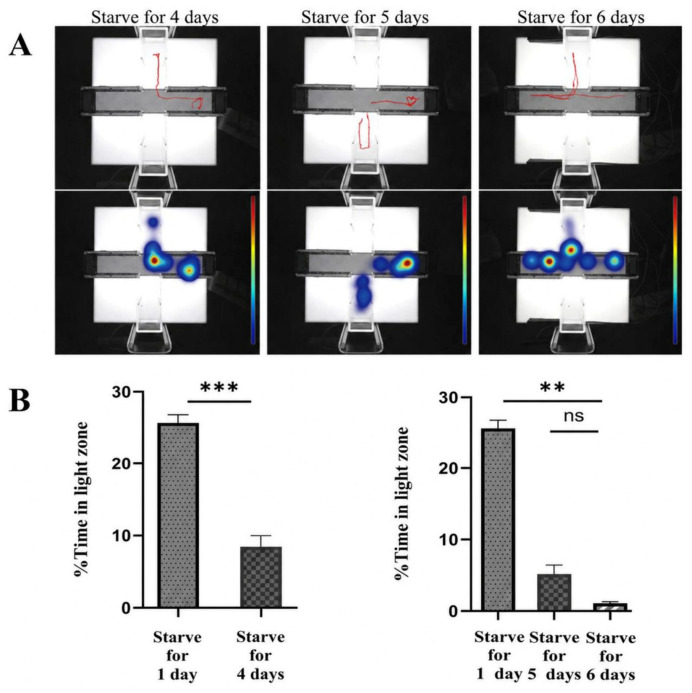
Starvation-associated behavioral responses of *P. trituberculatus* from 4 to 6 days. (**A**) Heatmap and movement trajectory; (**B**) percentage of time spent in the light arm. Asterisks in the figure represent *p* < 0.01 (**) and *p* < 0.001 (***) and ns: not significant.

**Figure 4:**
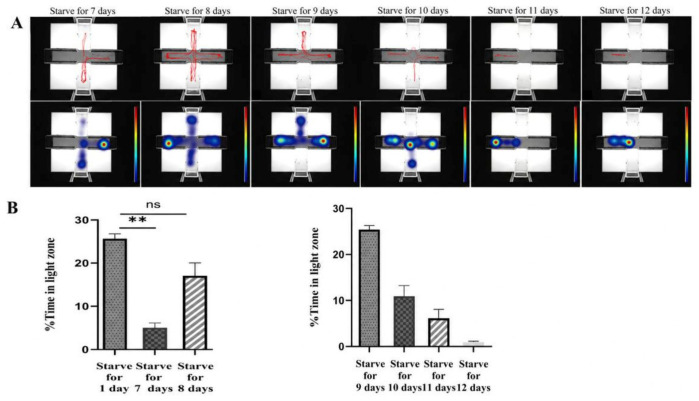
Starvation-associated behavioral responses of *P. trituberculatus* from 7 to 12 days. (**A**) Heatmap and movement trajectory; (**B**) percentage of time spent in the light arm. Asterisks in figures represent *p* < 0.01 (**) and ns: not significant.

During this period, the proportion of time spent in the light arm was significantly different from that of the behavioral reference group (*p* < 0.05), whereas the overall distance moved by *P. trituberculatus* did not change significantly. In addition, the time spent in the light arm increased again after 7 days of starvation (Figure 3) and recovered to a level similar to that of the behavioral reference group at 8 days of starvation. After 9 days of starvation, the time spent in the light arm gradually decreased again; after 10 days of starvation (Figure 4), the time spent in the light arm was nearly absent and then remained stable.

### 3.2. Open-Field Experiment

**Figure 5:**
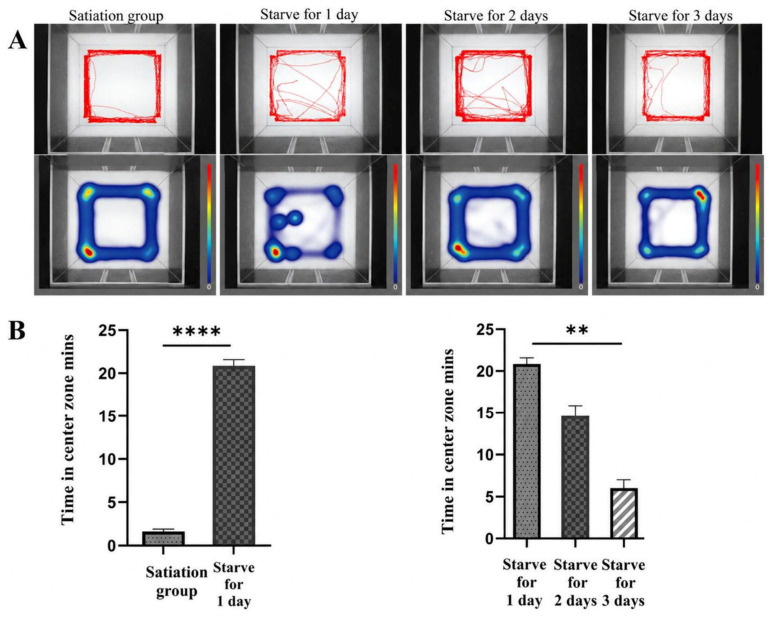
Starvation-associated behavioral responses of *P. trituberculatus* from 0 to 3 days. (**A**) Heatmap and movement trajectory; (**B**) time spent in the center zone. Asterisks in figures represent *p* < 0.01 (**) and *p* < 0.0001 (****).

The movement tracks and heat maps showed that crabs in the 0-day fed control group were mainly distributed in the peripheral zone. In the 1-day starved group, the time spent in the center zone increased significantly compared with the 0-day fed control group (*F*(1, 22) = 641.31, *p* = 9.13 × 10^−18^; Figure 5).

**Figure 6:**
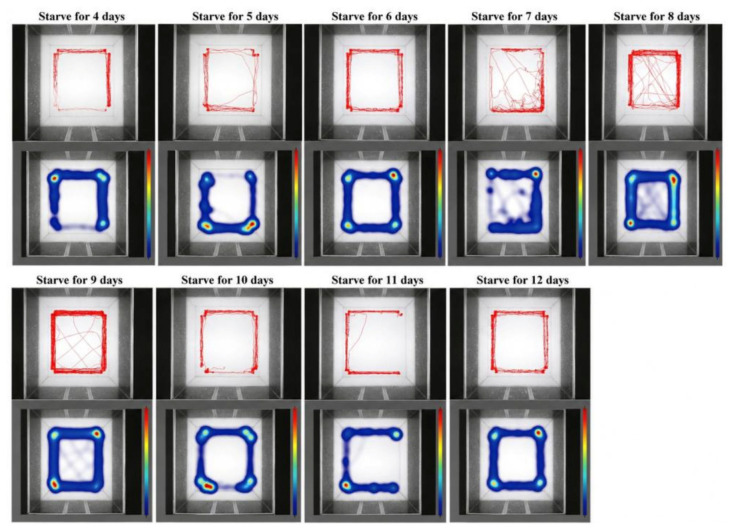
Starvation-associated behavioral responses of *P. trituberculatus* from 4 to 12 days: heatmaps and movement trajectories.

**Figure 7:**
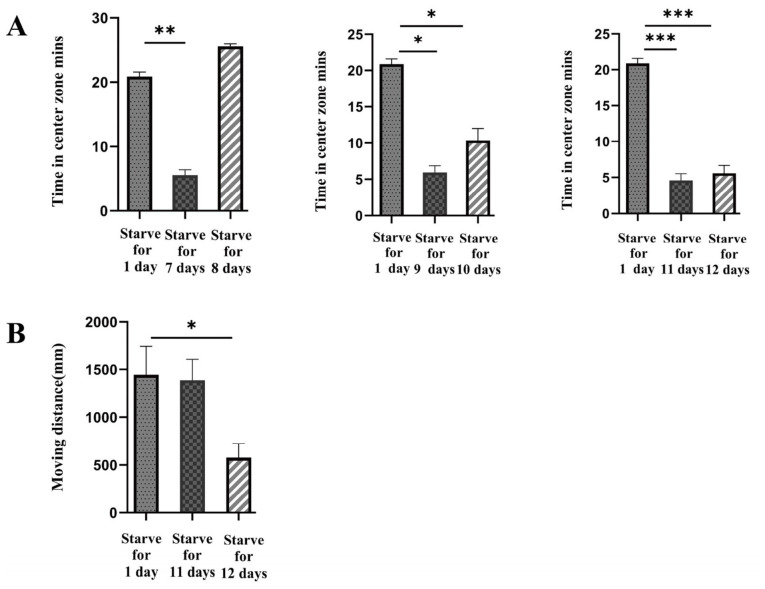
Starvation-associated behavioral responses of *P. trituberculatus* from 7 to 12 days. (**A**) Time spent in the center zone; (**B**) total distance moved among the 0-day fed control, 11-day starved, and 12-day starved groups. Asterisks in figures represent *p* < 0.05 (*), *p* < 0.01 (**), *p* < 0.001 (***).

After 3–6 days of starvation, the time spent by *P. trituberculatus* in the center zone gradually decreased. Compared with the 1-day starvation behavioral reference group, the 3-day starvation group showed a significant decrease in time spent in the center zone (*F*(1, 22) = 150.24, *p* = 2.64 × 10^−11^). After 7 days of starvation, time spent in the center zone increased and returned to a level similar to that of the behavioral reference group at 8 days. However, after 9 days of starvation, time spent in the center zone decreased again and nearly disappeared after 10 days. However, after 9 days of starvation, time spent in the center zone decreased again and nearly disappeared after 10 days (Figure 6 and Figure 7).

The open-field test and underwater light–dark cross-maze test showed consistent patterns of starvation-associated behavioral changes.

### 3.3. Effect of Starvation-Associated Behavioral Changes on Aggressive Behavior

**Figure 8:**
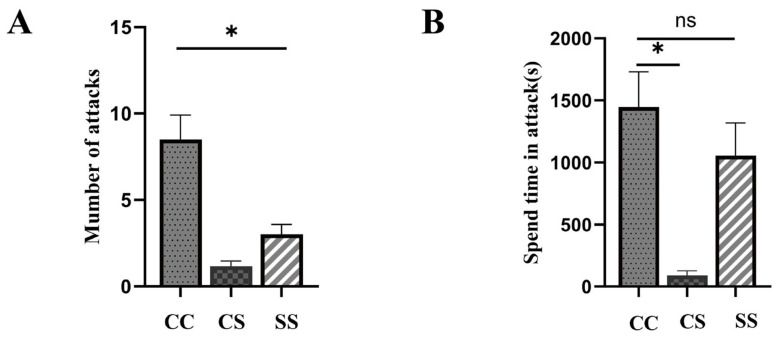
Attack frequency and attack duration in different pairing groups. (**A**) Attack frequency; (**B**) attack duration. CC, two 0-day fed control crabs paired together; SS, two starvation-treated crabs paired together; CS, one 0-day fed control crab paired with one starvation-treated crab. Asterisks in figures represent *p* < 0.05 (*), and ns: not significant.

Attack behavior differed among the three groups (Figure 8). Compared with the CC group, the SS group showed no significant difference in time spent in attack, but the number of attacks was significantly reduced (*p* < 0.05). In the CS group, both time spent in attack and the number of attacks were significantly lower than those in the CC group (*p* < 0.05).

### 3.4. Determination of 5-HT Concentration in Hemolymph

**Figure 9:**
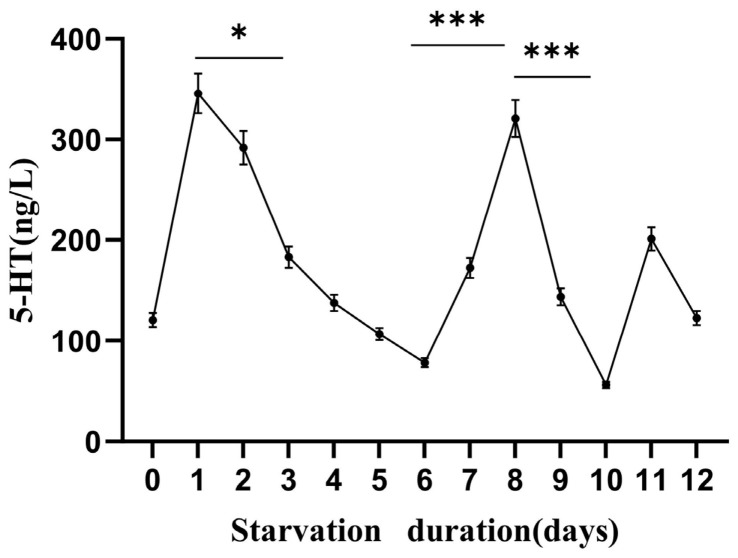
Changes in hemolymph 5-HT concentration across starvation durations. Asterisks in figures represent *p* < 0.05 (*) and *p* < 0.001 (***).

Compared with the 0-day fed control group, hemolymph 5-HT concentration increased in the 1-day starved group. It then decreased during 3–6 days of starvation, increased again during 7–8 days of starvation, and decreased after 9 days of starvation until reaching a relatively stable level (Figure 9). During the period when time spent in the light arm and center zone decreased, hemolymph 5-HT concentration also decreased significantly. When time spent in these zones increased, hemolymph 5-HT concentration increased, and it then decreased again during the later starvation period.

### 3.5. Expression of 5-HT1, 5-HT2, and DA1 in Different Tissues After Aggressive-Behavior Assays

**Figure 10:**
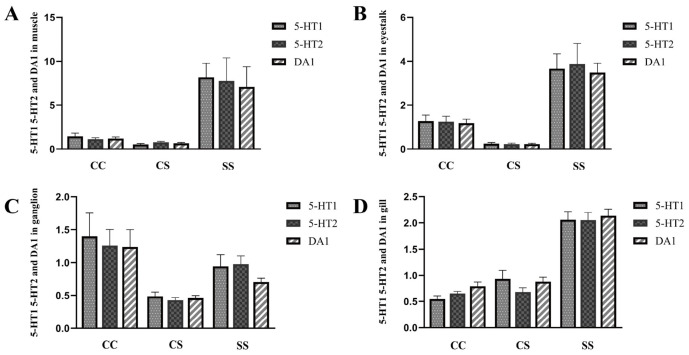
Expression levels of 5-HT1, 5-HT2, and DA1 in different tissues after aggressive-behavior assays. (**A**) Muscle; (**B**) eyestalk; (**C**) ganglion; (**D**) gill. CC, two 0-day fed control crabs paired together; SS, two starvation-treated crabs paired together; CS, one 0-day fed control crab paired with one starvation-treated crab.

After aggressive-behavior assays, 5-HT1, 5-HT2, and DA1 expression differed among tissues and pairing groups (Figure 10). In muscle tissue, 5-HT1, 5-HT2, and DA1 expression did not differ significantly between the CC and SS groups. However, 5-HT1 expression was significantly lower in the CC group than in the CS group (*p* < 0.05), whereas 5-HT2 and DA1 expression showed no significant differences among groups. In the eyestalk, 5-HT1, 5-HT2, and DA1 expression did not differ significantly between the CC and SS groups, but all three genes showed significantly higher expression in the CS group than in the SS group. No significant differences were observed between the CC and CS groups. In ganglion tissue, 5-HT1 and DA1 expression tended to be higher in the CS group than in the CC and SS groups, but these differences were not statistically significant. In gill tissue, 5-HT1, 5-HT2, and DA1 expression also tended to be higher in the CS group than in the other two groups, but no significant differences were detected among groups.

## 4. Discussion

Starvation-associated behavioral responses in *P. trituberculatus* changed dynamically across starvation durations. Dark-arm stay in the 0-day fed control group provided a behavioral baseline for interpreting starvation-associated changes in light-arm and center-zone stay. During the early starvation period, especially at 1–3 days, time spent in the light arm and center zone decreased, whereas total movement did not show a marked reduction at 1 day. This pattern suggests that early starvation-associated behavioral changes were not simply caused by general locomotor suppression [28].

Hemolymph 5-HT concentration changed in parallel with these behavioral changes. It decreased during the period when time spent in the light arm and center zone was reduced, increased again during the 7–8 day starvation period, and declined during the later starvation period. The increase at 7–8 days may be related to enhanced foraging motivation under prolonged food deprivation, which is consistent with previous observations that *P. trituberculatus* shows active food-searching behavior after several days without food [19]. Moderate hunger may also promote resource-seeking behavior in animals [29]. However, the present study measured hemolymph 5-HT concentration but did not directly manipulate serotonergic signaling; therefore, these associations should not be interpreted as causal.

After 9 days of starvation, reduced center-zone stay and reduced locomotor activity were observed. This late-stage behavioral suppression should not be interpreted simply as an anxiety-like state. Instead, it may reflect severe metabolic stress, reduced locomotor capacity, physiological exhaustion, or energy-conservation strategies under prolonged starvation. Because energy reserves, physiological condition, and locomotor capacity were not independently quantified, the behavioral changes observed during late starvation should be interpreted with caution. Future studies should include physiological indicators, such as energy-reserve measurements and locomotor-capacity assessments, to distinguish starvation-associated behavioral changes from starvation-induced exhaustion.

Starvation-associated behavioral states were also related to changes in aggressive behavior. Compared with the CC group, attack frequency was reduced in the SS group, and both attack frequency and attack duration were reduced in the CS group. These findings suggest that starvation may shift behavioral allocation away from energetically costly aggressive encounters. Starvation-driven behavioral prioritization has been reported previously [30], and such prioritization may help animals allocate limited energy to behaviors more directly related to immediate survival. However, the present data do not establish the causal mechanisms underlying the reduction in aggressive behavior.

The expression patterns of 5-HT1, 5-HT2, and DA1 differed among tissues and pairing groups after aggressive-behavior assays. Changes were more evident in muscle and eyestalk tissues, whereas most differences in ganglion and gill tissues were not statistically significant. These findings suggest that 5-HT1, 5-HT2, and DA1 expression may be involved in tissue-specific responses associated with starvation and aggressive-behavior contexts. Serotonergic and dopaminergic signaling systems are involved in behavioral regulation in invertebrates, including crustaceans [16,17]. Previous studies also suggest that 5-HT- and DA-related signaling may contribute to anxiety-like and aggression-related behavioral regulation, although their functions may differ among species [31,32].

The mechanistic interpretation of these molecular results remains limited. This study examined only 5-HT1, 5-HT2, and DA1 expression and did not provide a complete survey of serotonergic and dopaminergic receptor families in *P. trituberculatus*. In addition, monoamine synthesis-related genes, such as tryptophan hydroxylase, tyrosine hydroxylase, and DOPA decarboxylase, were not examined. Therefore, future studies should combine monoamine measurements, synthesis-related markers, receptor-family identification, and functional validation approaches, such as pharmacological manipulation or gene-functional assays, to clarify whether 5-HT and DA signaling pathways causally regulate starvation-associated behavioral changes and aggression in *P. trituberculatus*.

Although primer specificity was supported by single melting peaks and in silico PCR-predicted amplicon sizes, amplification efficiencies and Sanger sequencing validation of PCR products were not available in the present study. Future studies should include standard-curve-based efficiency testing and sequencing validation to further confirm primer specificity.

## Figures and Tables

**Table 1:** Primer sequences used for RT-qPCR analysis.

Primer Name	Primer Sequence (5′→3′)	Primer Information
*5-HTR1*-F	CGCCGCCTTCATCAGTTTGC	RT-qPCR primers
*5-HTR1*-R	GCCTGTGCCTTACGCTCCT	RT-qPCR primers
*5-HTR2*-F	GCAGCCATACTCGGACTTACGAC	RT-qPCR primers
*5-HTR2*-R	CGTCCAGCATGTCATTGCACT	RT-qPCR primers
*DA1*-F	GCAAACCTGTTCGTAGTGTCC	RT-qPCR primers
*DA1*-R	CAGGTTCACGATGGAAGCAG	RT-qPCR primers
RPL-F	GCACTGTCACCGATGACCTC	Internal reference primer
RPL-R	CCTTGCACCAGCAGAGTGTT	Internal reference primer

## Data Availability

The raw data supporting the conclusions of this article will be made available by the authors upon reasonable request.

## Supplementary Materials

The following supporting information can be downloaded at https://www.mdpi.com/article/10.3390/genes17060678/s1, Table S1: Stability of RPL expression across tissues and treatment groups; Table S2: Primer validation evidence summary; Table S3: Statistics an raw data.
